# Imaging diagnosis of classical and new pneumoconiosis: predominant reticular HRCT pattern

**DOI:** 10.1186/s13244-021-00966-y

**Published:** 2021-03-10

**Authors:** Akira Masanori

**Affiliations:** Department of Radiology, NHO Kinki-Chuo Chest Medical Center, 1180 Nagasone-cho, Kita-ku, Sakai City, Osaka 591-8555 Japan

**Keywords:** Pneumoconiosis, Asbestosis, HRCT, Metal lung disease

## Abstract

Our understanding of the manifestations of pneumoconioses is evolving in recent years. Associations between novel exposures and diffuse interstitial lung disease have been newly recognized. In advanced asbestosis, two types of fibrosis are seen, probably related to dose of exposure, existence of pleural fibrosis, and the host factor status of the individual. In pneumoconiosis of predominant reticular type, nodular opacities are often seen in the early phase. The nodular pattern is centrilobular, although some in metal lung show perilymphatic distribution, mimicking sarcoidosis. High-resolution computed tomography enables a more comprehensive correlation between the pathologic findings and clinically relevant imaging findings. The clinician must understand the spectrum of characteristic imaging features related to both known dust exposures and to historically recent new dust exposures.

## Key points

- The radiological pattern of known pneumoconiosis is changing in recent years.
- In advanced asbestosis, two types of fibrosis are seen on HRCT, probably related to dose of exposure, existence of pleural fibrosis, and the host factor status of the individual.
- Similar and different pathologic and radiologic findings are seen in each metal lung disease.
- The clinician must understand the spectrum of typical HRCT features related to dust exposures.

## Introduction

Pneumoconiosis is a classic disease, but exposures to asbestos and silica dust still affect workers. Asbestos and other harmful minerals have been used worldwide. Asbestos has been banned in the industrialized countries. Even now, there are many people in the world who are associated with mining, production, and uses of asbestos and other harmful minerals in the workplace. Professions and products that have risk of exposure to each of the pneumoconiosis are listed in Table 1.

**Table 1:** Professions and products that have risk of exposure to each of the pneumoconiosis

Agent	Professions and products
Asbestosis	Hard rock mining, construction, road work, tunneling, sandblasting, foundry work, glass manufacture, asbestos-cement products, asphalt floorings, floor tiles, asbestos textiles, asbestos paper products, friction materials
Talc pneumoconiosis	Paints, cosmetic powders, soapstone, ceramics, asphalt, shoe polish, roofing felts, rubber, fertilizer, refractory filler, paper, textiles
Aluminum pneumoconiosis	Refining of bauxite, aluminum smelting, abrasives, aluminum arch welders, aluminum powder, building materials, glass manufacture
Hard metal lung	Hard metal production, grinding, diamond polishing
Indium lung	Transparent electrodes for liquid crystal displays, touchscreens and solar cells, semiconductors

Classical pneumoconiosis is changing the typical pattern. The prevalence of severe and rapidly progressive pneumoconiosis is increasing [1]. The imaging features of metal-related lung disease, such as hard metal lung and aluminosis, have not been completely understood. Associations between novel exposures and diffuse interstitial lung disease or terminal airways disease have newly recognized [2, 3].

Correlation of high-resolution CT (HRCT) features with history of exposure is most important for the diagnosis of pneumoconiosis. HRCT features of predominant reticular type pneumoconiosis can mimic those of idiopathic interstitial pneumonias. In this article, we demonstrate pathology and HRCT findings of asbestosis, uncommon pneumoconioses, and newly recognized pneumoconioses. Compared to chest radiography, HRCT offers improved correlations between histopathologic and clinically relevant imaging findings [4–7].

## Asbestosis

In early asbestosis, the fibrotic process is limited to the walls of alveoli around the bronchioles. From this centrilobular position, fibrosis extends outward until it ultimately links adjacent bronchioles [8]. The fibrotic process affects the lower lobes of the lungs and may extent to the middle and upper lobes. In a study of the deposition and clearance of asbestos in rats using radioactive tracer techniques, autoradiographs of lung sections indicated that alveolar deposition was relatively uniform initially, that over a period of several months the uniform distribution changed to one in which fibers accumulated in foci that are mainly subpleural, and that these foci acted as centers for the development of nodular fibrosis [9].

**Fig. 1:**
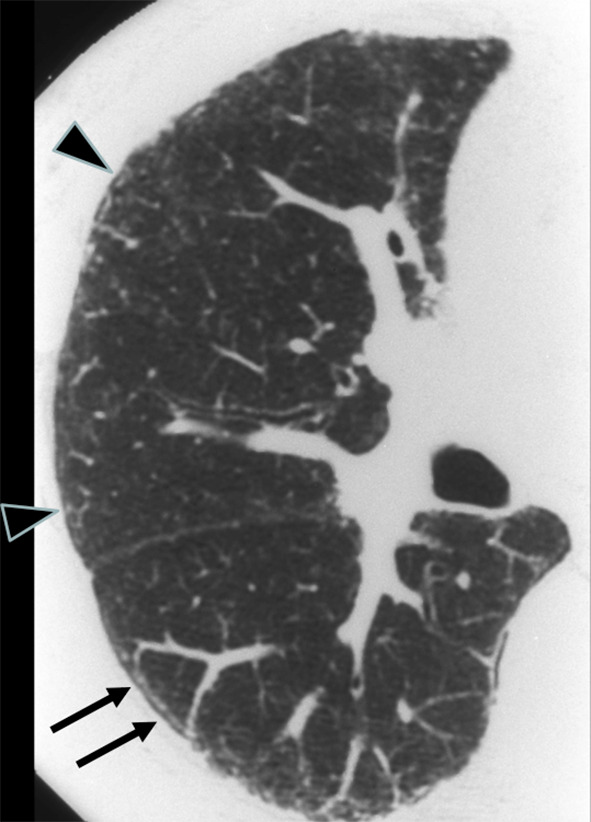
Subpleural dots and subpleural lines in asbestosis. Some dots are located a few millimeters from the pleura (arrows). In asbestosis, subpleural line is much closer to the pleural surface and the distance of the subpleural lines from the inner chest wall is 2 to 3 mm (arrowheads). The subpleural lines look like connected subpleural dots arranged along the inner chest wall

In the early stage of asbestosis, the HRCT findings include subpleural dot-like structures, subpleural lines, intralobular interstitial thickening, interlobular septal thickening, ground-glass opacities, and parenchymal bands [4–6]. CT-pathologic correlation shows that the subpleural dot-like structures correspond to peribronchiolar fibrosis with subsequent involvement of the alveolar ducts and that the subpleural lines correspond to peribronchiolar fibrosis combined with flattening and collapse of the alveoli. Subpleural dot-like structures arranged along the inner chest wall create subpleural lines (Fig. 1) [7].

**Fig. 2:**
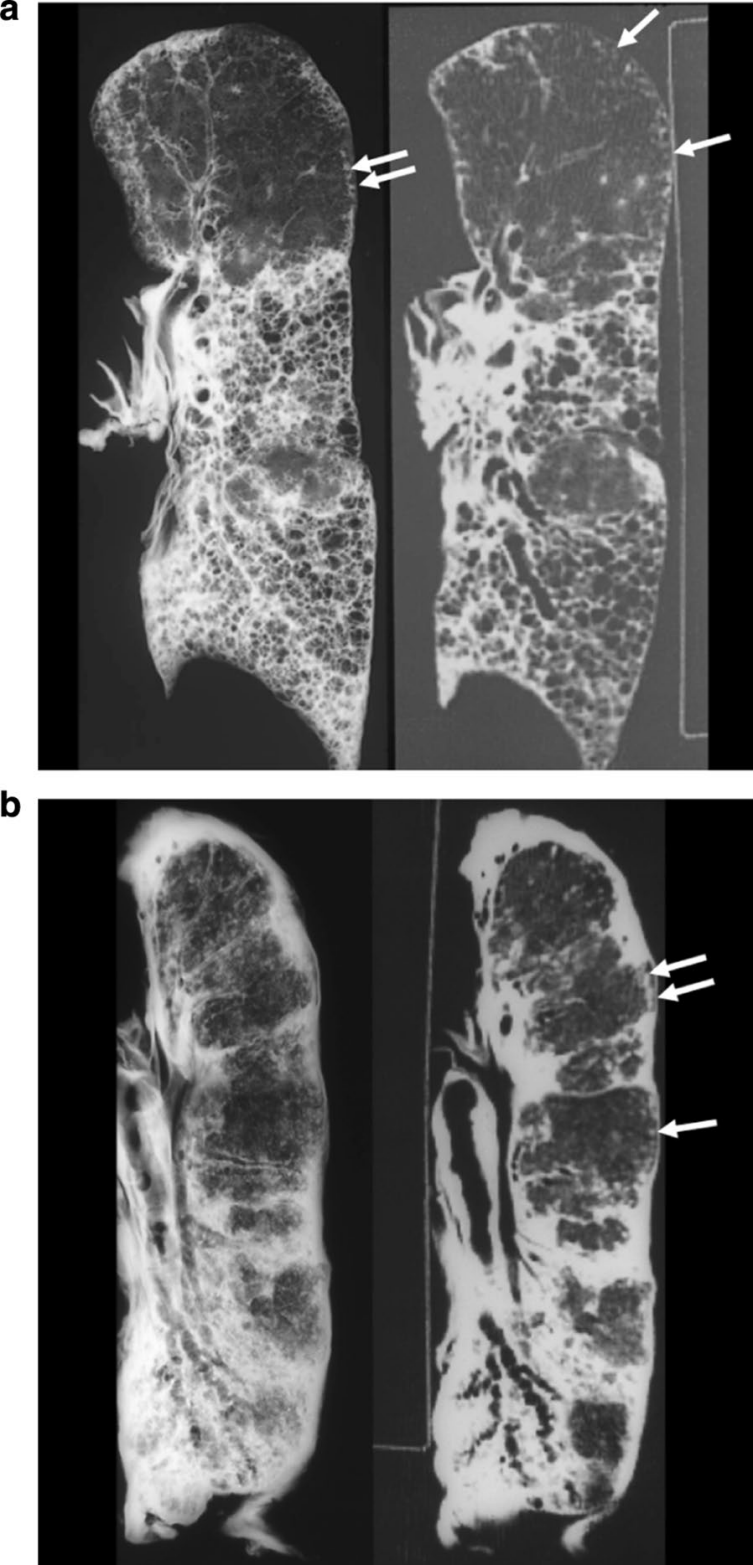
Two types of advanced asbestosis. **a** A honeycomb type of asbestosis. Postmortem low-kilovoltage radiograph and HRCT scan of the inflated and fixed left lung shows honeycombing in the lower two-thirds of the lung. In the subpleural zones of upper lung, dot-like lesions are seen (arrows). **b** An atelectatic fibrosis type of asbestosis. Postmortem low-kilovoltage radiograph and HRCT scan of the inflated and fixed left lung shows diffuse pleural thickening and consolidated area extending along the bronchovascular sheath. Traction bronchiectasis is seen in the consolidated area. Honeycombing is not seen radiologically. In the subpleural zones of upper lung, subpleural dots and subpleural lines are also seen (arrows)

In advanced asbestosis, there are two patterns of parenchymal abnormalities, i.e., honeycombing and atelectatic induration fibrosis (Fig. 2a, b) [10]. The frequency of honeycombing on HRCT scans is 17–32% [4, 11, 12]. Atelectatic induration fibrosis is characterized by a combination of collapse and collagenous fibrosis filling the alveolar lumens [8, 10]. The atelectatic induration type evolves much more rapidly and is related to high exposures. Atelectatic induration fibrosis is now rarely encountered in industrialized nations; however, such disease is likely to develop if asbestos dust control is neglected [8]. In a study, atelectatic induration fibrosis type showed 920,000 ± 1,360,000 asbestos fibers/g of dry lung, whereas honeycomb type showed 200,000 ± 490,000 asbestos fibers (*p* < 0.01) [13]. The characteristic HRCT finding is honeycombing, mimicking usual interstitial pneumonia (UIP). The HRCT findings of atelectatic induration fibrosis type are characterized by consolidation with loss of volume and traction bronchiectasis in the consolidated areas. Honeycombing is rare on the images. Pleural plaques, subpleural dots, and subpleural lines are present in both types. Diffuse pleural thickening is more frequent in the atelectatic induration fibrosis type than in the honeycomb type [13]. In patients with advanced disease, subpleural dot-like structures and subpleural lines are found in less severely involved regions of the pulmonary parenchyma [11].

In a cohort referred for diagnosis of an asbestos-related malignancy in the context of litigation, sixty-five cases with both adequate tissue sampling for histopathologic evaluation of asbestos and radiographic assessment of pulmonary fibrosis reported by B-reader who is a physician certified by the National Institute for Occupational Safety and Health (NIOSH) as demonstrating proficiency in classifying radiographs of the pneumoconioses were examined [14]. Of these, 24 cases were diagnosed as positive for asbestosis based on exposure and the presence ILO standard of profusion 1/0 or greater. The six cases of those 24 cases showed histologically proven asbestosis. The remaining 18 cases with B readings ≧ 1/0 showed interstitial fibrosis consistent with smoking-associated pulmonary fibrosis [14]. Another two cases showed histopathologic evidence of asbestosis but an ILO profusion of < 1/0. It may be possible that persons having many diseases other than asbestosis are included under asbestos-exposed persons with B readings ≧ 1/0.

The principal differential diagnosis of asbestosis is from idiopathic pulmonary fibrosis (IPF). Copley et al. [15] concluded that the HRCT pattern of asbestosis closely resembled that of UIP and differed markedly from that of nonspecific interstitial pneumonia (NSIP). They found that patients with asbestosis had coarser fibrosis than those with IPF but not when the analysis was confined to biopsy-proved UIP.

Cysts in asbestosis rarely exceed about 3 mm in diameter when present, but macrocystic honeycombing can be seen in asbestosis. Histopathologically, some cases of asbestosis resemble UIP, while others resemble fibrotic NSIP [8]. It is also said that the changes resemble those of NSIP, or more rarely UIP pathologically [16]. The fibroblastic foci that characterize the UIP pattern of fibrosing alveolitis are seldom observed in asbestosis [8].

The clinical progression is slow or has stabilized over time in asbestosis, as opposed to IPF. However, some of the past cases evolved much more rapidly, presumably related to high exposures.

In the study of pathologic and HRCT evaluation for 33 asbestos workers [17], 15 cases were pathologically diagnosed as asbestosis and 18 cases as various lung fibroses other than asbestosis. They concluded that on HRCT, subpleural lines were the only clue for the diagnosis of asbestosis. Mixed dust fibrosis and chronic interstitial pneumonia of unclassifiable histopathology, which are airway-centered, were included in the study. They may have centrilobular nodules, however, seldom show subpleural dot-like structures along with the pleural surface which create subpleural lines (Fig. 2). Moreover, there is a significant difference between IPF and asbestosis in pleural changes.

## Talc pneumoconiosis

**Fig. 3.**
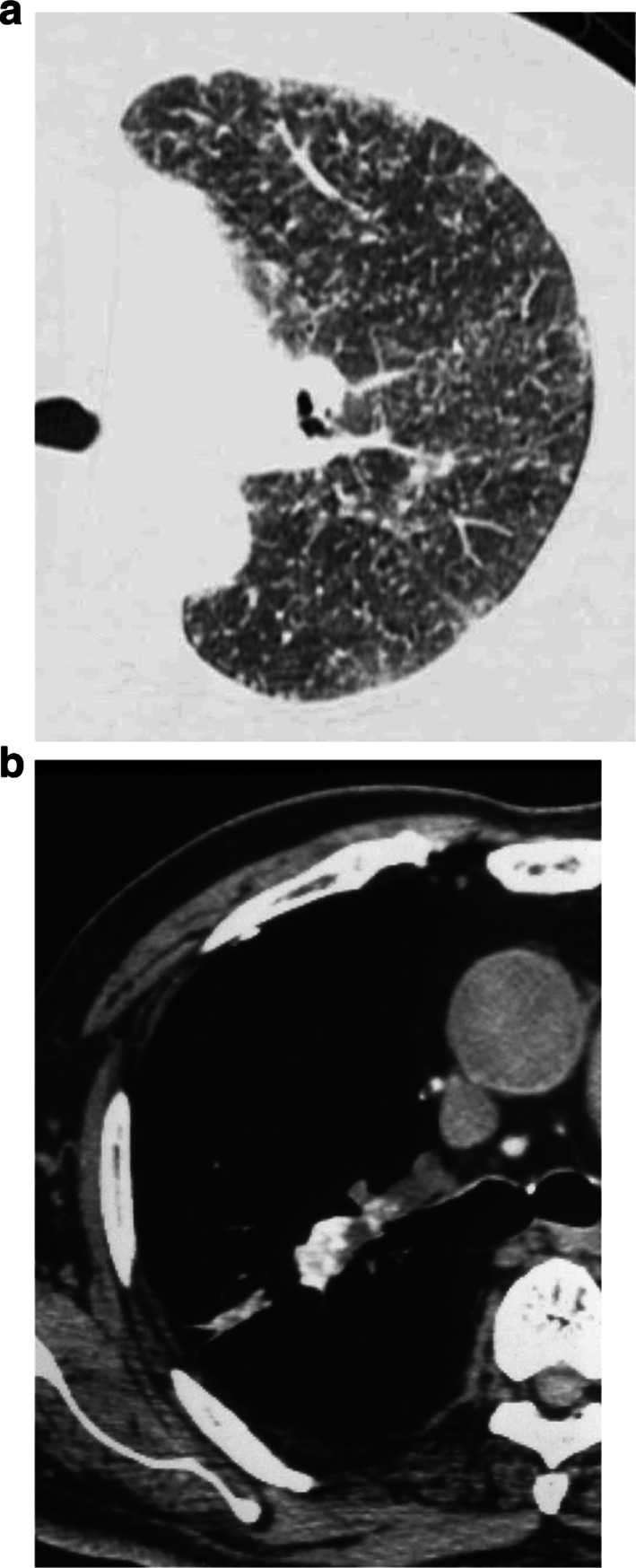
61-year-old man with inhalational talc pneumoconiosis employed in talc industry for 20 years. **a** HRCT scan shows diffusely distributed small nodules. They are separated from the pulmonary vein or the pleura at a distance of about 2–3 mm and are separated regularly from each other at a distance of about 2–3 mm. **b** CT scan at mediastinal setting of 61-year-old man with inhalational talc pneumoconiosis shows large opacity and lymph nodes containing high-attenuation material

High-resolution CT findings in talcosis from inhaled talc consist of diffuse small centrilobular nodules (Fig. 3a), ground-glass opacities, and heterogeneous conglomerate masses with high-attenuation areas consistent with talc deposition. The parenchymal abnormalities are diffuse but most severe in the upper and middle lung zones with relative sparing of the lung bases [18–21]. Conglomerated masses in silicosis usually involve the upper-lung zones, while in talcosis they are distributed throughout all lung zones [20, 21]. In talcosis observed in soapstone artisans, interlobular septal thickening was found in all patients, in addition to small centrilobular nodules (75%) and ground-glass opacity (67%) [22]. Slight lymph node enlargement with increased attenuation is visible in some cases of talcosis (Fig. 3b). Increased CT attenuation of lymph nodes and large opacities is caused by large numbers of talc particles [21].

CT findings in talcosis from injected talc include diffuse small nodules, perihilar conglomerated masses with areas of high attenuation, ground-glass opacities, and, particularly in patients with talcosis secondary to methylphenidate abuse, panacinar emphysema [19, 23, 24]. IV talcosis is usually hematogenous in distribution. The small nodules have a predominantly perivascular distribution. A case of pulmonary intravascular talcosis mimicking military tuberculosis has been reported [25].

## Metal-related lung diseases

Inhalation of metals and their fumes can induce a wide range of lung pathology, including granulomatous disease, giant cell interstitial pneumonitis (GIP), chemical pneumonitis, interstitial fibrosis, airways disorders, and cancer. Metals such as aluminum, cobalt, copper, and indium induce pulmonary fibrosis. Metal-induced pulmonary fibrosis has some similarities.

## Aluminum pneumoconiosis

**Fig. 4:**
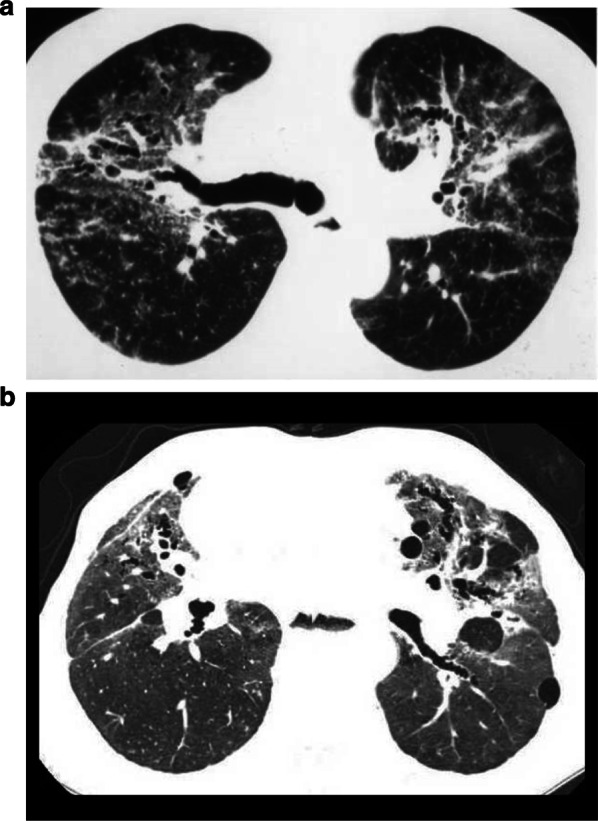
HRCT scans of pulmonary aluminosis. **a** 58-year-old man with pulmonary aluminosis. HRCT scan of pulmonary aluminosis mimicking sarcoidosis. Ground-glass opacities, small nodular opacities, and traction bronchiectasis are seen predominantly around the bronchovascular bundles. Nodules are located in both centrilobular and paralobular regions. **b** 52-year-old man with pulmonary aluminosis. HRCT shows traction bronchiectasis and ground-glass opacity predominantly in the upper lungs. Multiple bullae and centrilobular nodules are also seen

Aluminum lung is characterized by diffuse interstitial fibrosis mainly located in the upper and middle lobes of the lung. In the early stages of aluminosis, the HRCT findings are characterized by small rounded and ill-defined centrilobular opacities mainly in the upper lobes [26, 27]. Alveolar septal fibrosis or centrilobular nodules under the resolution of HRCT scans create ground-glass opacity. In addition to centrilobular nodules, the nodular pattern in aluminosis includes nodules in lymphatic distribution mimicking sarcoidosis, probably reflecting a granulomatous lung reaction (Fig. 4a, b).

In advanced stages, it is characterized by subpleural bullous emphysema with an increased risk of spontaneous pneumothorax [28]. The HRCT appearances of aluminum pneumoconiosis may be variable and include nodular, reticular, and upper lung fibrosis pattern (Fig. 4a, b) [28]. Conglomerated masses mimicking silicosis are thought to be affected by exposure to silica other than aluminum dust. Interstitial lung disease in corundum (aluminum oxide) abrasive workers can be accelerating silicosis or mixed dust fibrosis.

Increased attenuation of mediastinal lymph nodes with histologically proven aluminum storage can be seen on CT scans [29].

## Hard metal pneumoconiosis

Exposure to hard metal causes asthmatic reaction, hypersensitivity pneumonitis, and pulmonary fibrosis [30]. Patients typically present with hard metal interstitial lung disease after 10–12 years of exposure, but the disease can occur in as little as 2 years [31]. GIP is the classic pathology of cobalt related interstitial lung disease. GIP is characterized by a chronic interstitial pneumonia with fibrosis that is typically bronchiolocentric [32].

**Fig. 5:**
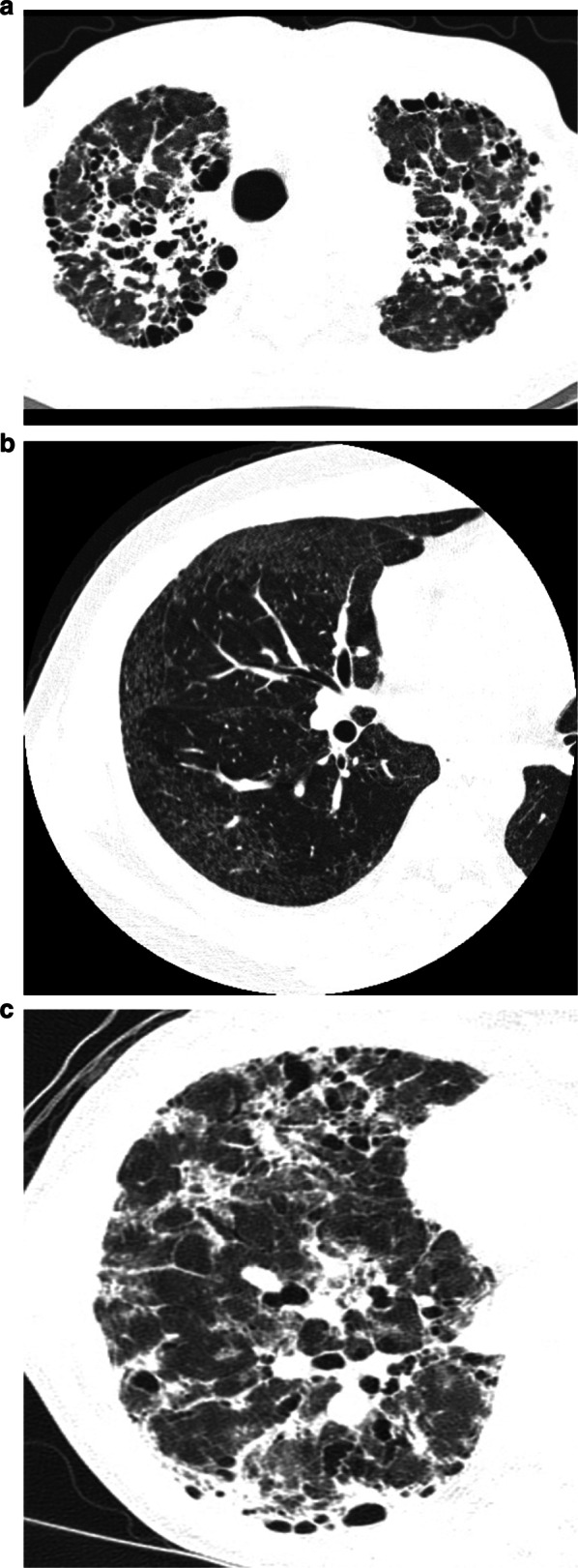
HRCT scans of heard metal pneumoconiosis. **a** 62-year-old man with hard metal pneumoconiosis. Upper-lung predominant fibrosis in hard metal pneumoconiosis. HRCT scan shows prominent interstitial thickening, irregular peribronchovascular thickening, and traction bronchiectasis in upper lung zones. **b** 32-year-old man with hard metal pneumoconiosis. Early stage of hard metal pneumoconiosis. HRCT scan shows ground-glass opacities and centrilobular nodules predominantly in the peripheral portions. **c** 53-year-old man with hard metal pneumoconiosis. HRCT scan shows patchy and irregular ground-glass opacity and traction bronchiectasis diffusely distributed in the lung. Bullae are seen in the subpleural region.

The HRCT appearance of hard metal interstitial lung disease may be variable and may mimic sarcoidosis, NSIP, UIP, and fibrotic hypersensitivity pneumonitis [28, 33–37]. As for the predominant distribution of parenchymal abnormalities, both upper predominant (Fig. 5a) and lower predominant distribution are reported to the same degree. The major HRCT findings are ground-glass opacities. The ground-glass opacities show diffuse, patchy, or lobular distribution. Reticular opacities and small nodular opacities are also seen. The small nodules are distributed mainly in centrilobular location [28, 35–37]. Some patients show perilymphatic distribution, mimicking sarcoidosis [33, 36]. In some cases with hard metal pneumoconiosis, the predominant HRCT finding is centrilobular nodules. The centrilobular nodules on HRCT correspond to the histologic finding of bronchiolocentric fibrosis [28]. Ground-glass centrilobular nodules and air trapping are the predominant findings in cobalt-related ILD with a HP pattern [38].

In the early stages of hard metal pneumoconiosis, the HRCT scan shows ground-glass opacities and centrilobular nodules (Fig. 5b) [39]. Traction bronchiectasis and architectural distortion are seen in advanced cases. Clustered cystic lesions are also seen and consist of traction bronchiectasis and bronchiolectasis, and bullae (Fig. 5c). Consolidation is a rare finding, seen in a fatal case [28]. Peripheral cystic spaces are reported in some cases [28, 33, 40]. Lymph node enlargement and spontaneous pneumothorax have been reported [30].

**Fig. 6.**
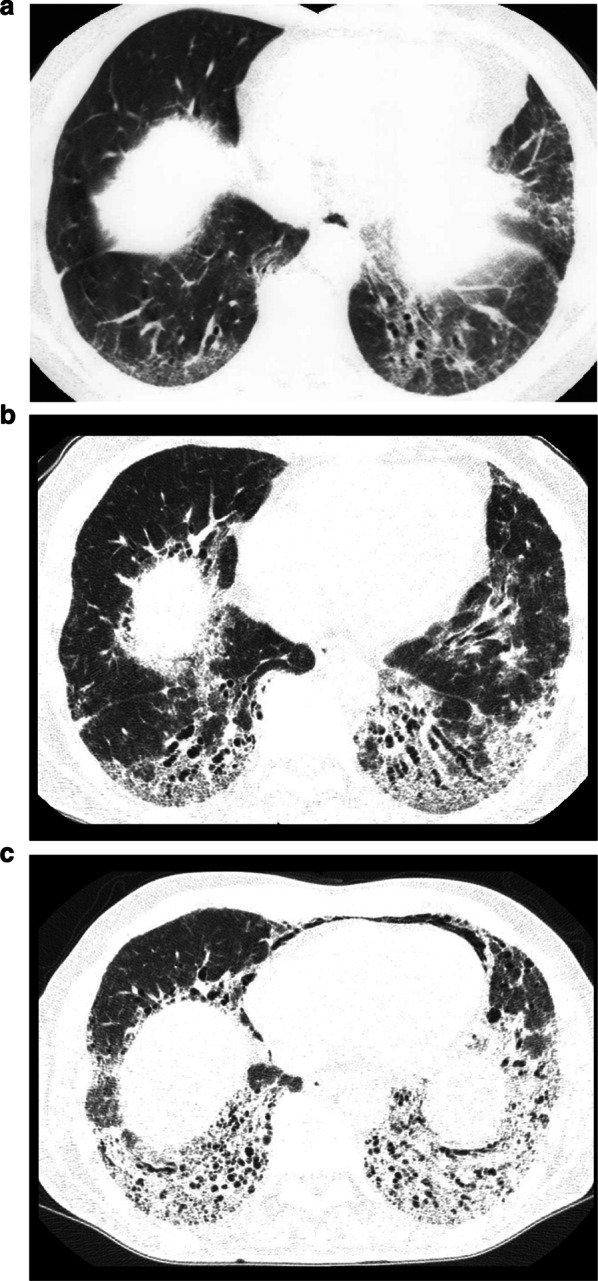
59-year-old man with hard metal pneumoconiosis. Initial HRCT scan (**a**) shows scattered areas of ground-glass attenuation associated with fine reticulation and mild traction bronchiectasis. HRCT obtained 3 years later (**b**) reveals increase in parenchymal abnormalities in extent, prominent reticulation, and progression of traction bronchiectasis. HRCT obtained 5 years later (**c**) shows dense increased parenchymal opacities with traction bronchiectasis

Progression of fibrosis in hard metal lung disease is very different from case to case (Fig. 6). The progression is slow in some cases, and others rapidly deteriorate [28, 31, 41]. Ground-glass opacities are reported to improve following cessation of exposure to hard metal and treatment [35]. Patients with fibrosis are less likely to experience disease remission.

## Indium lung disease

Workplace exposure to indium compounds including indium oxide and indium-tin oxide causes several lung diseases; pulmonary fibrosis, emphysema and pulmonary alveolar proteinosis (PAP). Ten clinical cases of lung disease in indium workers from three countries (Japan, United States, and China) had been reported [42, 43]. Among the reported cases, those with shorter diagnostic latency had findings more consistent with PAP, while cases with longer diagnostic latency had findings more consistent with interstitial lung disease. Two patients initially diagnosed with PAP had radiographic progression to fibrosis over several years [43]. Amata et al. reported that radiographic interstitial changes can be reduced in indium workers by cessation of exposure to indium, whereas emphysematous lesions can progress among those with a history of heavy exposure [44]. An advanced case of indium lung disease with severely progressive emphysema has been reported [45].

**Fig. 7:**
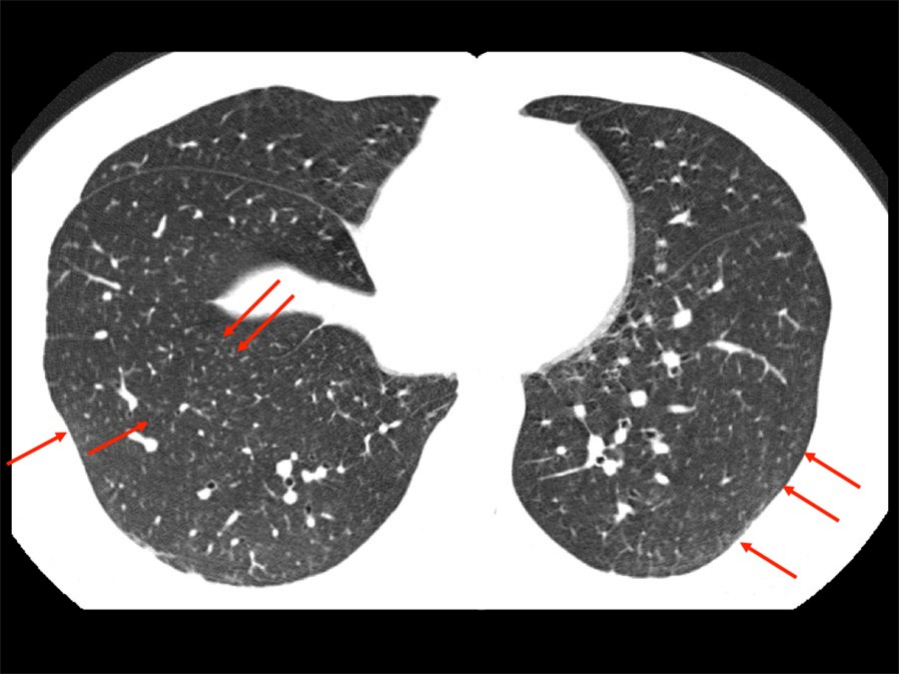
HRCT scan of a 34-year-old man with indium lung. Tiny centrilobular nodules (arrows) are scattered in both lungs

In cases with interstitial lung disease, the major HRCT findings are ground-glass opacities and/or centrilobular opacities (Fig. 7) [43]. The parenchymal abnormalities show diffuse, upper or mid-lung predominance, and sometimes lower-lung predominance. Interstitial opacities with volume reduction and traction bronchiectasis in the upper lungs are reported [42]. Paraseptal emphysema, small cysts at both apices and subpleural honeycomb can be seen [42].

## Nylon flock worker’s lung

In nylon flock worker’s lung, histopathologic evaluation commonly shows a NSIP pattern with lymphocytic bronchiolitis with peribronchovascular interstitial lymphoid infiltrates. Other secondary histological features that were variably present include diffuse alveolar damage (DAD), bronchiolitis obliterans with organizing pneumonia (BOOP), and desquamative interstitial pneumonia (DIP) [46–48]. HRCT appearances reflect these histopathologic findings.

The most common imaging findings in flock workers’ lung are ground-glass opacities and micronodules predominantly distributed in the centrilobular region. The parenchymal abnormalities are diffuse, patchy, or predominantly show a basal and peripheral pattern. Reticular opacities, traction bronchiectasis, septal thickening, and consolidation are also seen [46–49]. In some cases, HRCT shows extensive basal-predominant ground-glass opacity similar to that seen in NSIP or DIP. In advanced stages, HRCT shows subpleural-predominant reticular abnormalities associated with honeycombing and traction bronchiectasis similar to that of UIP [49].

Flock workers’ lung shows profuse micronodules and more extensive ground-glass opacity compared to IPF. These HRCT features are included under non-UIP patterns that are considered to be inconsistent with IPF in the 2011 American Thoracic Society/European Respiratory Society/Japanese Respiratory Society/Latin American Thoracic Association guidelines [50].

## Newly recognized pneumoconioses

In addition to indium lung disease and flock workers’ lung, recent studies have linked new causative occupational and environmental agents with both airways disease and parenchymal lung disease [2]. Representative examples are shown in Table 2. The last four entities are not discussed in the text of the manuscript. Further studies on each entity are needed.

**Table 2:** Newly recognized pneumoconioses

Agent	Causative agent	Characteristic histopathology	CT findings
Indium lung	Indium-tin oxide	Interstitial fibrosis, PAP, emphysema	Ground-glass opacities, centrilobular nodules, traction bronchiectasis, crazy-paving appearance, LAA
Nylon flock worker’s lung	Nylon fibers	NSIP with lymphocytic bronchiolitis, DIP	Diffuse micronodular opacities, patchy consolidation, honeycombing
Flavor worker’s lung (Popcorn worker’s lung)	Diacetyl	BO	Mosaic attenuation (air trapping)
World-trade center lung	Complex amalgam, fiberglass, fly ash, silica, asbestos, etc	Asthmatic bronchiolitis, BO, AEP, sarcoid-like granulomatosis	Mosaic attenuation (air trapping), peripheral consolidation, diffuse micronodules
Nanoparticles	Carbon nanotubes, etc	Interstitial fibrosis	Patchy ground-glass opacities, centrilobular nodules, traction bronchiectasis
Ardystil syndrome	Acramin-FWN	OP	Peripheral predominant patchy consolidation

PAP, pulmonary alveolar proteinosis; NSIP, nonspecific interstitial pneumonia; DIP, desquamative interstitial pneumonia; BO, bronchiolitis obliterans; AEP, acute eosinophilic pneumonia; OP, organizing pneumonia

## Conclusion

Asbestosis has two types of fibrosis, probably related to dose of exposure, existence of pleural fibrosis, and the host factor status of the individual. Metal-related lung diseases have similarities and differences irrespective of kinds of metal. Some of the radiologic and pathologic findings of pneumoconiosis resemble those of idiopathic interstitial pneumonias. Dust exposure can cause pathologic and radiologic changes indistinguishable from IPF. It is postulated that many types of dust exposures can increase risk of developing IPF [51]. HRCT reflects pathologic changes of pneumoconiosis. HRCT would enable a more comprehensive correlation between the pathologic findings and the clinically relevant imaging findings. Further study of HRCT of pneumoconiosis is warranted.

## Data Availability

The data are published in the article.

## Abbreviations

BOOP: Bronchiolitis obliterans organizing pneumonia
CT: Computed tomography
DAD: Diffuse alveolar damage
DIP: Desquamative interstitial pneumonia
GIP: Giant cell interstitial pneumonia
HP: Hypersensitivity pneumonitis
HRCT: High-resolution computed tomography
ILO: International Labour Organization
IPF: Idiopathic pulmonary fibrosis
NSIP: Non-specific interstitial pneumonia
PAP: Pulmonary alveolar proteinosis
UIP: Usual interstitial pneumonia
